# Sheng Mai San Regulating the Oxidative Stress and Mitochondrial Damage to Alleviate Liver Injury in Heat Stress Rats

**DOI:** 10.3390/ani16091391

**Published:** 2026-05-02

**Authors:** Qian Ma, Jiaqi Dong, Xiaosong Zhang, Rong Yang, Yanming Wei

**Affiliations:** 1 College of Veterinary Medicine, Gansu Agricultural University, Lanzhou 730070, China; 107332214022@st.gsau.edu (Q.M.); 18893811442@163.com (J.D.); yrong1005@126.com (R.Y.); 2 College of Veterinary Medicine, China Agricultural University, Beijing 100091, China; zhangxsgs@163.com

**Keywords:** Sheng Mai San, heat stress, liver injury, oxidative stress, mitochondrial damage

## Abstract

Sheng Mai San (SMS), a traditional Chinese medicine formula for treating qi and yin deficiency, is widely used in the management of conditions such as cardiovascular diseases and heatstroke. However, its role in mitigating heat stress (HS)-induced liver injury remains underexplored. In this study, a rat model of HS was established under high-temperature and high-humidity conditions, and SMS was administered as an intervention. The pharmacodynamic effects of SMS were comprehensively evaluated through histopathological examination, detection of heat shock protein 70 (HSP70) and heat shock protein 90(HSP90) expression, and analysis of liver function biomarkers (AST, ALT). Meanwhile, oxidative stress indicators were measured using biochemical assay kits (GSH, SOD, CAT, MDA, T-AOC), and transmission electron microscopy was employed to observe mitochondrial ultrastructure, thereby assessing the protective effects of SMS on hepatic oxidative stress and mitochondrial damage induced by HS. In vitro, BRL-3A cells were cultured, subjected to HS, and treated with SMS. Cell viability was assessed using the CCK-8 assay, and changes in mitochondrial reactive oxygen species (ROS) levels, mitochondrial permeability transition pore (MPTP) opening, and mitochondrial membrane potential (MMP) were evaluated using fluorescent probes. The results showed that SMS effectively restored HS-induced histopathological damage in rat liver tissues, reduced serum AST and ALT levels, and downregulated the mRNA expression of HSP70 and HSP90 in liver tissues. Meanwhile, SMS strengthened the hepatic antioxidant system by increasing the levels of GSH, SOD, T-AOC, and CAT, while decreasing MDA content. In vitro experiments confirmed that SMS increased the viability of BRL-3A cells, reduced ROS production, improved MPTP opening/closing regulation, and stabilized MMP. This study provides a clinical reference for its application in treating HS-related conditions in humans and animals.

## Simple Summary

Sheng Mai San (SMS) was investigated for its protective effects against heat stress (HS)-induced liver injury in rats and BRL-3A cells. Results showed that SMS reduced liver tissue damage, lowered serum AST and ALT levels, and decreased HSP70/HSP90 expression. It also enhanced antioxidant capacity by increasing GSH, SOD, CAT, and T-AOC while reducing MDA. In vitro, SMS improved cell viability, mitochondrial function, and reduced oxidative stress. This study supports SMS as a potential treatment for HS-related liver injury.

## 1. Introduction

Heat stress (HS) induced by high temperatures presents a significant challenge for both human health and animal husbandry production [1]. Solar radiation and wind speed can swiftly elevate the ambient temperature above the thermoneutral zone for livestock and poultry [2,3]. resulting in a total heat load that exceeds their inherent heat dissipation capacity and thus triggering HS [4]. When HS coincides with high environmental humidity, the consequences of high temperatures on livestock and poultry intensify significantly because their evaporative heat loss is diminished. As one of the most common stressful events in animal production, HS can result in decreased productivity, compromised animal welfare, lowered fertility rates, as well as heightened susceptibility to diseases and increased mortality [5]. According to reports, in 2002, a devastating HS event led to the deaths of over 30,000 dairy cows in California, USA. In Iowa, more than 4000 beef cattle died due to HS, and in California, over 700,000 poultry suddenly perished as a result of HS [6]; Between 2002 and 2003, the U.S. beef and poultry industries experienced annual economic losses of approximately $369 million and $128 million, respectively, due to HS [7], Despite implementing HS mitigation strategies in 2010, the U.S. pig farming industry still incurred economic losses exceeding $900 million during the summer [8]. Notably, HS not only impairs animal production performance but also causes severe damage to multiple organs, among which the liver is particularly vulnerable. Addressing the detrimental impacts of HS on livestock and poultry health, as well as on the prevention and control of animal diseases, and elucidating the mechanisms responsible for the initiation and progression of HS, holds immense theoretical, economic, and societal importance for the discovery of effective pharmacological treatments and preventive strategies to mitigate its effects.

The liver stands as a pivotal organ within the body, exerting a fundamental role in various processes, including substance metabolism, detoxification, and immune function. HS has the potential to impair liver function or even cause liver failure, affecting nearly 5% of patients who receive long-term HS treatment or have pre-existing liver diseases (e.g., chronic hepatitis, non-alcoholic fatty liver disease) and may thus suffer from severe, life-threatening liver damage. This damage can result in hepatocyte degeneration and necrosis, as well as bile duct obstruction, among others [8]. Despite this, the precise mechanisms that drive the occurrence and progression of these effects remain subjects of ongoing debate and controversy in the scientific community [9,10,11]. Research has shown that when HS occurs, the body is in a state of elevated body temperature and high metabolism. The liver, serving as a pivotal metabolic organ, experiences an even higher core temperature. An excessively elevated core temperature within the liver directly results in the disruption and denaturation of cytoskeletal proteins in hepatocytes, damaging the hepatocytes and causing liver dysfunction or even failure. The degree of liver injury is closely related to the occurrence, development, and prognosis of HS [11,12]. Numerous studies propose that the mechanism underlying HS arises from heightened energy metabolism and oxygen demand in body tissues during periods of heat exposure [13,14,15]. Chronic exposure to excessive reactive oxygen species (ROS) continually assaults vital enzymes involved in nucleic acid, protein, Chronic exposure to excessive ROS continually assaults vital enzymes involved in nucleic acid, protein, and lipid metabolism within cells, inhibiting the biological activity of antioxidant enzymes [16]. Mitochondria act as the main energy-supplying organelles in cells and are responsible for the majority of endogenous ROS generation under both physiological and pathological conditions [17,18]. As primary targets of oxidative stress [19], these organelles are highly susceptible to oxidative injury, which further impairs their normal functions and exacerbates cellular damage.

Sheng Mai San (SMS) originates from “Yi Xue Qi Yuan”, authored by Zhang Yuansu, a renowned physician of the Jin Dynasty. It is a classic prescription in Traditional Chinese Medicine (TCM) for nourishing both qi and yin where “qi” refers to vital energy maintaining normal physiological functions, and “yin” refers to nutrient substances that moisten and nourish the human body. This prescription is composed of *Panax ginseng* C.A. Mey.g, *Ophiopogon japonicus* (L. f) Ker-Gawl., and *Schisandra chinensis* (Turcz.) Baill, whose main pharmacologically active components include ginsenosides, ophiopogonin, and schizandrin [20]. The active components of *Panax ginseng* (*ginsenosides*) can regulate energy metabolism and tonify vital energy, while *Ophiopogon japonicus* (*ophiopogonin*) nourishes yin and generates fluids, and *Schisandra chinensis* (*schisandrin*) calms the heart and enhances antioxidant capacity. These components work synergistically to exert pharmacological effects. The combined use of these three herbs exerts a synergistic effect, which is reflected in regulating energy metabolism, enhancing antioxidant capacity, and improving body function, consistent with the TCM effects of invigorating qi, promoting fluid generation, consolidating yin, and calming the heart [21]. In modern clinical practice, SMS is commonly used to treat critical illnesses, including heatstroke, heart failure, liver failure, and shock. Additionally, it has exhibited promising therapeutic outcomes in managing chronic diseases like chronic bronchitis, tuberculosis, and diabetes [22,23]. Nevertheless, there have been limited reports regarding its protective role against liver damage caused by HS. In this study, in vivo and in vitro models of HS were established to comprehensively and systematically investigate the underlying mechanisms of HS onset and progression using multiple parameters, including histopathology and liver function. On this basis, SMS was administered to heat-stressed rats and BRL-3A cells as an intervention. Transmission electron microscopy, fluorescent probes, and flow cytometry were employed to assess oxidative stress levels, as well as changes in mitochondrial morphology and function. Through this systematic investigation, this study aims to elucidate the protective mechanisms of SMS against HS-induced liver injury, thereby establishing a solid research foundation for its potential clinical application.

## 2. Materials and Methods

### 2.1. Reagents

N-Acetylcysteine (NAC, CAS:616-91-1), Reactive Oxygen Species Assay Kit, (ROS, S0033S), Enhanced Mitochondrial Membrane Potential Assay Kit, Mitochondrial Permeability Transition Pore Assay Kit (MPTP, C2003) were purchased from Bi yun Tian Biotechnology (Shanghai, China); Kits for alanine aminotransferase (ALT, C009-2-1), aspartate aminotransferase (AST, C010-2-1), superoxide dismutase (SOD, A001-3-2), malondialdehyde (MDA, A003-1-2), reduced glutathione (GSH, A006-2-1), catalase (CAT, A007-1-1), and total antioxidant capacity (T-AOC, A015-2-1) were purchased from Nanjing Jiancheng Bioengineering Institute (Nanjing, China).

### 2.2. Preparation of SMS

In accordance with the standard decoction preparation method of traditional Chinese medicine, 24 g of the compound formula (9 g of *Panax ginseng* C.A. Mey., 9 g of *Ophiopogon japonicas* (Thunb.) Ker-Gawl., and 6 g of *Schisandra chinensis* (Turcz.) Baill.) were crushed and passed through a 20-mesh sieve. Ten times the amount of water was added, followed by a 30 min immersion and a 60 min decoction. The decoction was filtered through four layers of gauze, and the residue was subjected to a second extraction with eight volumes of water for 40 min. After the second extraction, the combined filtrates were centrifuged at 3000 r/min for 15 min to remove insoluble matter. The supernatant was concentrated under reduced pressure at 60 °C using a rotary evaporator until a relative density of 1.0 g/mL was reached, and was then freeze-dried under vacuum to obtain the SMS test drug. The extraction yield was 57.67%. The dried extract was sealed and stored at low temperature until subsequent experimental use [24,25].

### 2.3. Animals and Ethics

#### 2.3.1. Establishment of HS Rat Models

Forty-eight male SPF-grade Sprague-Dawley rats aged 4–6 weeks and weighing 180 ± 20 g were used in this study. The animals were housed at 23 ± 1 °C and 45 ± 5% relative humidity under a 12 h light/dark cycle, with free access to standard chow and water. After a 7-day acclimatization period, the rats were randomly assigned to a control group and five heat stress groups corresponding to 0, 3, 6, 9, and 12 h after the final heat exposure, with eight rats in each group. Heat stress was induced by exposing the rats to 38 ± 1 °C and 75 ± 5% relative humidity for 2 h daily from 10:00 to 12:00 for 7 consecutive days. Except during the heat exposure period, the rats were kept under normal housing conditions. Control animals were maintained at 23 ± 1 °C and 45 ± 5% relative humidity throughout the experiment. Samples were collected at the indicated time points after the final heat exposure for model evaluation.

#### 2.3.2. SMS Intervention Treatment

SD rats were divided into six groups (*n* = 8) and received intragastric administration 2 h before HS each day. The control group (Control) and the HS group (HS) were administered normal saline daily, while the treatment groups received the corresponding doses of the test drugs. A positive control group was set up with NAC at a dose of 150 mg/kg [26]. The control group was maintained at an ambient temperature of 23 ± 1 °C and a relative humidity of 45 ± 5%. The HS group and all treatment groups were exposed to 2 h of heat stress for 7 consecutive days. After the last heat stress exposure, all groups were allowed to recover for 6 h before sample collection. The dosage of SMS was calculated using the formula Dr = (Dh/W) × F, where Dr is the rat dose, Dh is the human clinical dose derived from the *Pharmacopoeia of the People’s Republic of China* [27], W represents the assumed human body weight (60 kg), and F is the human-to-rat dose conversion factor (6.3). The high (SMS-H, 5.04 g/kg), medium (SMS-M, 2.52 g/kg), and low (SMS-L, 1.26 g/kg) dose groups corresponded to 0.5, 1, and 2 times the clinical dose, respectively. After the experiment, all rats were anesthetized by intraperitoneal injection of 10% chloral hydrate at a dose of 0.5 mL/100 g body weight. Blood samples were collected from the abdominal aorta using anticoagulant-free blood collection tubes. Liver samples were collected under sterile conditions, with 3 liver samples from each group used for fixation, and the remaining samples were stored at −80 °C.

#### 2.3.3. Histopathological Analysis

Eight rat livers were collected from each treatment group (Control, HS, SMS-L, SMS-M, SMS-H, and NAC groups). The collected livers were washed with saline to remove blood and fixed with 10% neutral formaldehyde. After fixation, samples were dehydrated via graded ethanol (70%, 80%, 95%, 100%; 15 min per step), cleared twice with xylene (15 min each), embedded in paraffin wax (melting point 56–58 °C) at 60 °C for 2 h, and sectioned into 5 μm slices. Sections were baked at 60 °C for 30 min, followed by H&E staining (hematoxylin for 8 min, 1% hydrochloric acid-ethanol differentiation for 30 s, eosin for 2 min), then dehydrated, cleared, and mounted with neutral gum. Finally, images were captured using the Olympus DP-71 system.

Histological evaluation of liver tissue damage was performed under a light microscope, and the degree of liver injury was graded according to the criteria of hepatocyte degeneration, necrosis, inflammatory cell infiltration, and bile duct dilation (graded as 0–4 points: 0 = no damage, 1 = mild damage, 2 = moderate damage, 3 = severe damage, 4 = extremely severe damage). Each section was evaluated independently by two pathologists who were blinded to the experimental groups to ensure objectivity.

#### 2.3.4. Transmission Electron Microscopy Observation

The electron microscope samples of liver tissue stored at 4 °C were taken and refixed with 1% osmium tetroxide. Acetone dehydration step by step, dehydrating agent concentration gradient is 30%, 50%, 70%, 80%, 90%, 95%, 100% (100% concentration change 3 times). The dehydrated samples were successively subjected to dehydrating agent and 812 epoxy resin penetrants in ratios of 3:1, 1:1, and 1:3, with each step lasting 30 to 60 min. The adequately penetrated sample blocks were then embedded, and ultra-thin sections approximately 50 nm thick were prepared using an ultrathin slicer. After floating on the surface of the groove liquid, the samples were transferred onto a copper mesh. They were first stained with uranyl acetate, followed by lead citrate staining at room temperature for 15–20 min. The samples were then observed using a JEM-1400 PLUS transmission electron microscope (JEOL, Japan).

### 2.4. Cell Experiments

#### 2.4.1. Cell Culture and HS Model in BRL-3A Cells

The BRL-3A rat liver cell line was obtained from Qingqi Biotechnology Development Co. (Shanghai, China). BRL-3A cells were cultured in complete DMEM containing 1% penicillin-streptomycin-amphotericin B, 10% fetal bovine serum, and 89% DMEM at 37 °C in a 5% CO_2_ incubator. Cells were passaged when they reached 70% confluence.

After 24 h of culture, BRL-3A cells were divided into a control group and an HS group with rewarming time points at 0, 3, 6, 9, and 12 h. The HS model group was incubated at 43 ± 0.5 °C in 5% CO_2_ for 2 h. Following HS, the cells were transferred to a 37 °C, 5% CO_2_ incubator and rewarmed for the respective time points.

#### 2.4.2. SMS Intervention and Grouping

After 24 h of culture, the cells were treated with SMS (at concentrations of 400, 800, and 1600 µg/mL) or NAC (at a concentration of 5 mM) for an additional 24 h. Following this intervention, all groups except the control group were subjected to HS in a 43 ± 0.5 °C, 5% CO_2_ incubator for 2 h. After modeling was completed, the cells were placed back in a 37 °C, 5% CO_2_ incubator for a recovery period of 9 h. Subsequently, the samples were collected and stored for further analysis.

#### 2.4.3. Measurement of Cell Viability

The cell density was adjusted to 7.5 × 10^4^ cells/mL, and 100 μL of the single-cell suspension was seeded into each well of a 96-well plate. After 24 h of adherence culture, the old medium was discarded, and 100 μL of CCK-8 working solution was added. After 1 h of incubation at 37 °C in 5% CO_2_, the OD value of each well was measured at 450 nm using a microplate reader. Each group included 5 parallel replicates, with the CCK-8 working solution used as the blank control. Cell viability for each group was calculated according to the specified formula.

#### 2.4.4. Cytomorphological Observation

The cell culture and heat stress model in BRL-3A cells was performed according to the procedures described in Section 2.4.1. The morphology of cells in each treatment group was observed and photographed using an OLYMPUS CKX53 inverted microscope at 200× magnification under bright-field imaging mode. Cells were washed twice with phosphate-buffered saline (PBS) before observation, and images were collected at the predetermined time points of 0, 3, 6, 9, and 12 h.

#### 2.4.5. Fluorescent Probe Assay

BRL-3A cells were seeded in 6-well plates. After 24 h of adherence culture, the cells were subjected to different treatments and washed three times with PBS. Subsequently, 1 mL of DCFH-DA probe, or JC-1 probe, or Calcein-AM probe was added, and the cells were incubated at 37 °C in a 5% CO_2_ incubator in the dark for 20 min. The cells were washed three times with PBS to remove any unloaded probe, and 1 mL of serum-free medium was added. Fluorescent images were then acquired using a fluorescence microscope at an excitation wavelength of 488 nm and an emission wavelength of 525 nm, with three parallel replicates per group.

#### 2.4.6. Flow Cytometry

BRL-3A cells were seeded in 6-well plates. After 24 h of adherence culture and treatment, the old medium was discarded, and the cells were washed once with PBS. They were then digested with 400 μL of EDTA-free trypsin per well. The cells were incubated at 37 °C with 5% CO_2_ for 3 min, and 400 μL of DMEM medium was added to stop the digestion. The cells were then collected and centrifuged at 1000 r/min for 5 min at room temperature. After discarding the supernatant, the cells were resuspended in 500 μL of 1:3000 DCFH-DA diluent. The cells were incubated with DCFH-DA at 37 °C in the dark under 5% CO_2_ for 20 min, with gentle mixing every 3–5 min to facilitate uniform probe loading and sufficient contact with the cells. After incubation, the cells were washed three times with serum-free culture medium and centrifuged at 1000 r/min for 5 min to remove residual extracellular DCFH-DA. The fluorescence signal was then detected by flow cytometry at an excitation wavelength of 488 nm and an emission wavelength of 525 nm.

### 2.5. Real-Time Quantitative Polymerase Chain Reaction (qPCR)

To extract total RNA from the samples, the TransZol Up kit was used. The RNA was reverse transcribed into first-strand cDNA using the TransScript^®^II Green One-Step qPCR SuperMix according to the manufacturer’s instructions. Subsequently, real-time PCR was performed to determine the mRNA expression levels of HSP70 and HSP90. All primers were designed and synthesized by Shanghai Sangon Biotech Co., Ltd (Shanghai, China). (Table S1). The PCR reaction was performed in a 20 μL volume containing cDNA template, SYBR Green mix, and forward and reverse primers. Amplification conditions were as follows: pre-denaturation at 95 °C for 30 s, followed by 40 cycles of denaturation at 95 °C for 5 s, annealing at 60 °C for 30 s, and extension at 72 °C for 30 s. Relative gene expression was calculated using the 2^−ΔΔCt^ method with GAPDH as the internal reference gene.

### 2.6. Determination of Serum AST and ALT

The detection methods for aspartate aminotransferase (AST) and alanine aminotransferase (ALT) in each group of rats are as follows: For serum sample detection, blood is first collected, and the collected blood samples are allowed to stand at room temperature for 30 min, then centrifuged at 2200 rpm at 4 °C for 10 min using a centrifuge (YG16, Shanghai, China). After centrifugation, the upper serum is aspirated to prepare rat serum samples. For cell sample detection, after the completion of cell culture, the cell culture is collected and centrifuged at 2200 rpm at 4 °C, and the upper cell supernatant is aspirated. Finally, the levels of AST and ALT in the serum and cell supernatant were detected by enzyme-linked immunosorbent assay (ELISA) using a microplate reader (Spectra Max 384, Molecular Devices, San Jose, CA, USA) in strict accordance with the instructions of the AST and ALT biochemical detection kits.

### 2.7. Determination of Oxidative Stress Index

The levels of CAT, SOD, GSH, T-AOC, and MDA in the rat serum and cell supernatant were all detected in accordance with the method described in Section 2.6. Their contents were strictly measured in accordance with the instructions of the corresponding detection kits by enzyme-linked immunosorbent assay (ELISA).

### 2.8. Statistical Analysis

Statistical analyses were conducted using SPSS 26.0, including independent samples *t*-tests and one-way ANOVA. Data are presented as mean ± standard error. Graphs were generated using GraphPad Prism 9. A *p*-value of < 0.05 was considered statistically significant.

## 3. Results

### 3.1. Evaluation of HS-Induced Liver Injury Model in Rats

**Figure 1:**
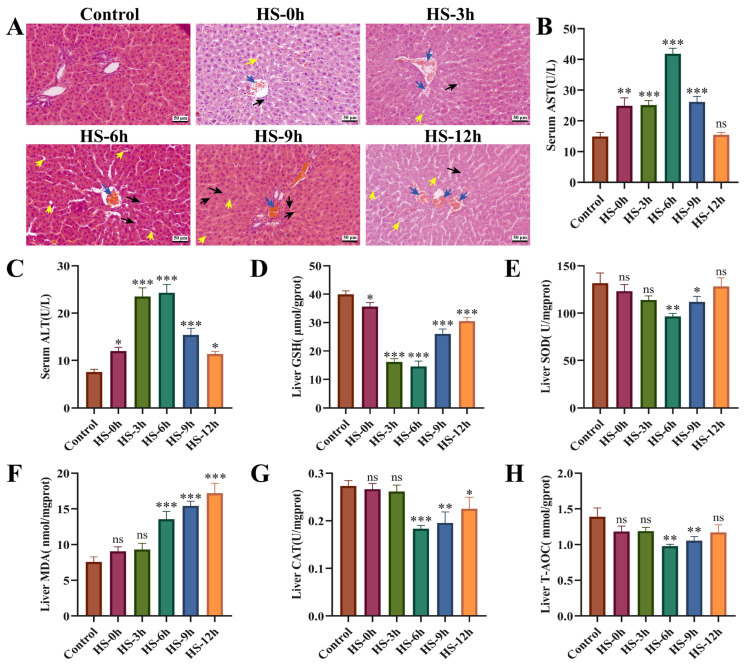
Evaluation of HS-induced liver injury model in rats. (**A**) Effects of different recovery times after HS on pathological changes in rat liver (400×). (**B**,**C**) Effects of different recovery times after HS on the expression of AST and ALT in rat serum (*n* ≥ 5/group). (**D**–**H**) Effects of different recovery times after HS on the expression of GSH, SOD, MDA, CAT and T-AOC in rat liver (*n* ≥ 5/group). Compared with the control group, ns, not significant, * *p* < 0.05, ** *p* < 0.01, *** *p* < 0.001. Note: Blue arrows indicate central vein and hepatic sinusoidal stasis; black arrows represent inflammatory cell infiltration; yellow arrows represent hepatocyte vacuolar degeneration.

The histopathological results indicated that the livers of heat-stressed rats exhibited varying degrees of damage at different rewarming time points, with the most severe damage occurring between 6 and 12 h. This damage was primarily characterized by changes such as congestion of the central vein and hepatic sinusoids, infiltration of inflammatory cells, and cellular vacuolar degeneration (Figure 1A). The biochemical results indicated that, compared to the control group, serum AST and ALT levels gradually increased with rewarming time following HS, peaking at 6 h before declining between 9 and 12 h (Figure 1B,C). In terms of oxidative stress-related indices, liver GSH levels significantly decreased (*p* < 0.05) from 0 to 12 h after the cessation of HS. SOD, CAT, and T-AOC levels began to decline significantly (*p* < 0.05) at 6 h post-HS, while MDA levels showed a marked increase (*p* < 0.01) at the same time (Figure 1D–H). These findings suggest that HS-induced liver injury primarily occurs between 6 and 12 h after the cessation of HS.

### 3.2. Pharmacodynamic Evaluation of SMS on HS-Induced Liver Injury in Rats

**Figure 2:**
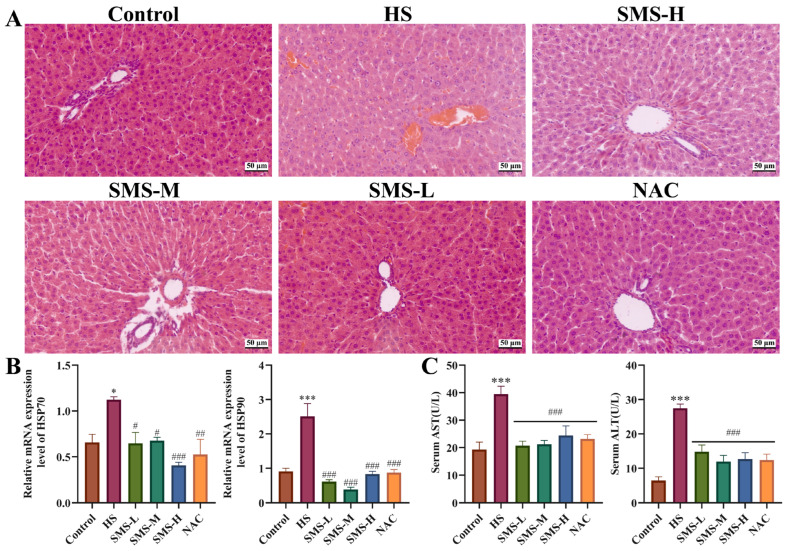
Pharmacodynamic evaluation of SMS on HS-induced liver injury in rats. (**A**) Effects of SMS and NAC on the histopathology change in the liver of heat-stressed rats (*n* = 3/group). (**B**) Effects of SMS and NAC on the mRNA expression of HSP70 and HSP90 in the liver (*n* ≥ 3/group). (**C**) Effects of SMS and NAC on the expression of AST and ALT in the serum (*n* ≥ 5/group). Compared with the control group, * *p* < 0.05, *** *p* < 0.001; compared with the HS group, # *p* < 0.05, ## *p* < 0.01, ### *p* < 0.001.

This study investigated the protective effects of SMS against HS-induced liver injury in rats by assessing histopathological changes, liver function markers, and the expression of heat shock proteins. Representative pathological alterations in the HS group, including diffuse congestion, vacuolar degeneration, and inflammatory cell infiltration, were markedly ameliorated following SMS treatment (Figure 2A). Compared with the control group, rats subjected to HS exhibited significantly elevated serum levels of AST and ALT, as well as increased mRNA expression of HSP70 and HSP90 in liver tissue (*p* < 0.05) (Figure 2B,C). Notably, these abnormalities were substantially reversed by intervention with SMS or NAC.

### 3.3. Effect of SMS on Oxidative Stress and Mitochondrial Damage in the Liver of Heat-Stressed Rats

**Figure 3:**
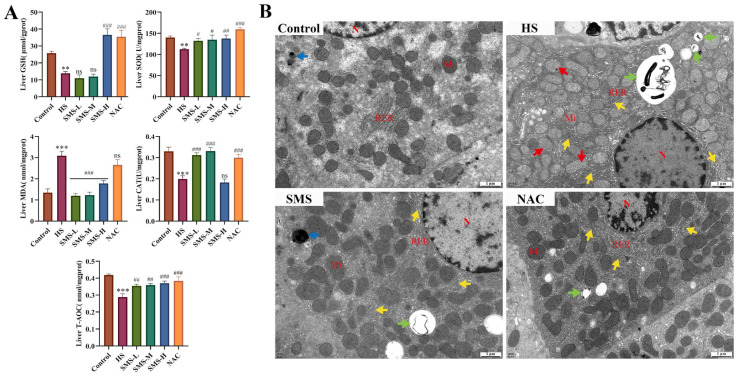
Effect of SMS on oxidative stress and mitochondrial damage in the liver of heat-stressed rats. (**A**) Effects of SMS and NAC on the expression of GSH, SOD, MDA, CAT and T-AOC in the liver (*n* ≥ 5/group). (**B**) Ultrastructure of the liver (*n* = 3/group, 15,000×), “N” represent nucleus, “RER” represent rough endoplasmic reticulum, “Mi” represent mitochondrion, red arrows represent mitochondrial swelling, green arrows represent autophagy, yellow arrows represent RER dilation, blue arrows represent secondary lysosome. Compared with the control group, ** *p* < 0.01, *** *p* < 0.001; compared with the HS group, ns, not significant, # *p* < 0.05, ## *p* < 0.01, ### *p* < 0.001.

Mitochondria, as the primary site of ROS production, are closely linked to oxidative stress. To investigate the protective mechanism of SMS against HS-induced liver injury, this study employed transmission electron microscopy to observe the ultrastructure of rat liver tissue and assessed oxidative stress-related parameters. The results showed that compared with the control group, the levels of GSH, SOD, CAT, and T-AOC in the liver of heat-stressed rats were significantly decreased (*p* < 0.01), whereas the MDA content was markedly increased (*p* < 0.001). Following intervention with SMS, all parameters were significantly ameliorated (Figure 3A).

Ultrastructural observation revealed that in the HS group, the majority of hepatocyte nuclei exhibited obvious shrinkage and chromatin aggregation. Meanwhile, the majority of cytoplasmic mitochondria were swollen, accompanied by decreased mitochondrial cristae, and partial mitochondrial fusion was observed. After the intervention with SMS, the rough endoplasmic reticulum in hepatocytes was mildly dilated; mitochondrial structure was well-preserved with abundant and neatly arranged cristae, and autophagic vacuoles were visible. Similarly, in the NAC positive control group, the rough endoplasmic reticulum was mildly dilated, mitochondrial morphology and structure were intact and regular, and a small number of autophagic vacuoles were observed in the cytoplasm (Figure 3B).

### 3.4. Evaluation of HS-Induced BRL-3A Cells Model

**Figure 4:**
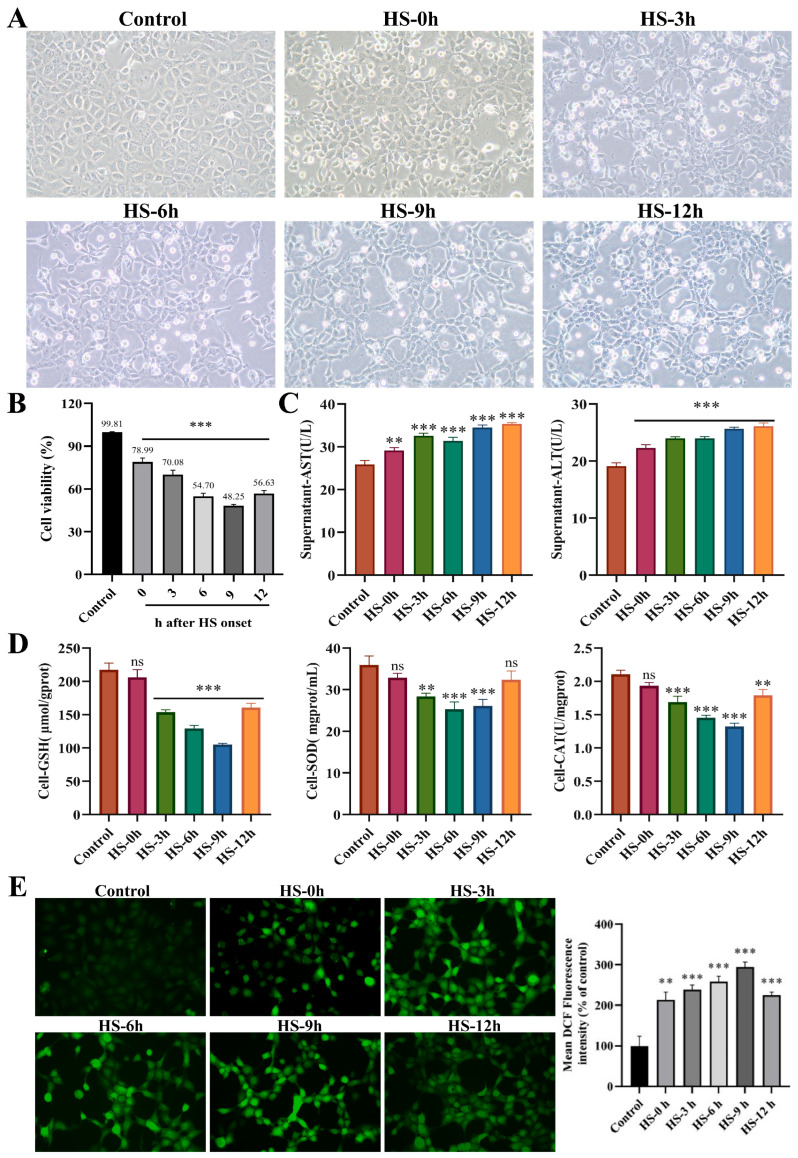
Evaluation of HS-induced BRL-3A cells model. (**A**) Effects of different recovery times after HS on the morphological changes in BRL-3A cells (200×, *n* = 3). (**B**) Effects of different recovery times after HS on the cellular activity of BRL-3A cells (*n* ≥ 3/group). (**C**) Effects of different recovery times after HS on the expression of AST and ALT in the supernatants of BRL-3A cells (*n* ≥ 3/group). (**D**) Effects of different recovery times after HS on the expression of GSH, SOD and CAT in BRL-3A cells (*n* ≥ 3/group). (**E**) Effects of different recovery times after HS on the content of released ROS in BRL-3A cells detected by DCFH-DA probe (*n* ≥ 3/group). Compared with the control group, ns, not significant, ** *p* < 0.01, *** *p* < 0.001.

The effects of different rewarming times after HS on BRL-3A cells were evaluated by assessing morphological changes, cell viability, liver injury markers, and oxidative stress indicators. Morphological analysis revealed that cell damage progressively worsened as rewarming time extended after HS. The number of dead suspended cells increased at 0 h, while adherent cells exhibited shrinkage, a decreased refractive index, and a scarcity of dividing nuclei. From 3 to 12 h, the number of dead suspended cells increased significantly, with cells appearing wrinkled and deformed, with unclear structural outlines and indistinct nuclear structures, and cells were diffusely distributed (Figure 4B). HS significantly reduced the viability of BRL-3A cells, reaching a minimum of 48.25% at 9 h of rewarming (*p* < 0.05). After 12 h of rewarming time, cell viability showed a slight rebound but remained significantly lower than that of the control group (*p* < 0.05) (Figure 4A). Biochemical results showed that AST and ALT levels in the cell supernatants increased progressively with rewarming time and were both significantly higher than those in the control group (*p* < 0.01) (Figure 4C).

Furthermore, beginning at 3 h after HS, the levels of GSH, SOD, and CAT in BRL-3A cells exhibited a continuous downward trend with prolonged rewarming time, remaining below normal levels and reaching their lowest values at 9 h (*p* < 0.001) (Figure 4D). HS induced a significant increase in ROS release in BRL-3A cells, which peaked at 9 h of rewarming (*p* < 0.001) and then slightly decreased at 12 h (Figure 4E). Collectively, these results indicate that HS-induced injury in BRL-3A cells progressively worsens with extended rewarming time, reaching its maximum severity at 9 h after HS.

### 3.5. Pharmacodynamic Evaluation of SMS on HS-Induced BRL-3A Cells Injury

**Figure 5:**
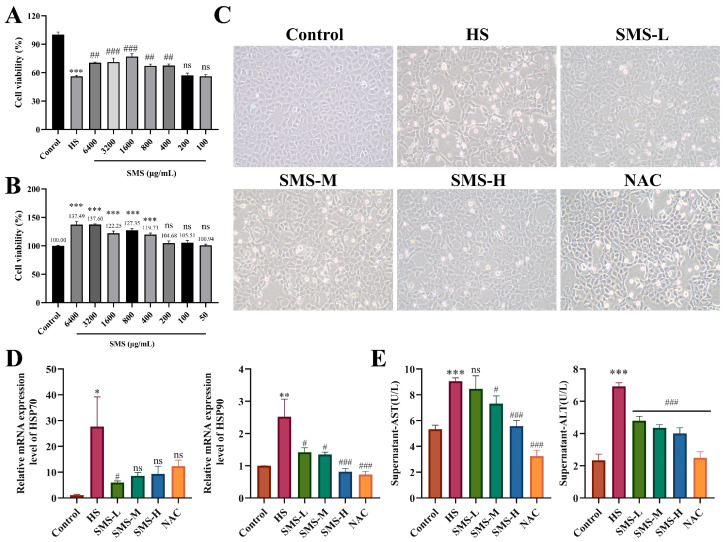
Pharmacodynamic evaluation of SMS on HS-induced BRL-3A cell injury. (**A**) Cytotoxicity of SMS on BRL-3A cells (200×, *n* ≥ 3/group). (**B**) Effects of SMS and NAC on the cell viability of BRL-3A cells subjected to HS (*n* ≥ 3/group). (**C**) Morphological changes in SMS and NAC on BRL-3A cells after HS treatment (*n* = 3/group). (**D**) Effects of SMS and NAC on the mRNA expression of HSP70 and HSP90 in BRL-3A cells (*n* ≥ 3/group). (**E**) Effects of SMS and NAC on the expression of AST and ALT in the supernatants of BRL-3A cells (*n* ≥ 3/group). Compared with the control group, ns, not significant, * *p* < 0.05, ** *p* < 0.01, *** *p* < 0.001; compared with the HS group, ns, not significant, # *p* < 0.05, ## *p* < 0.01, ### *p* < 0.001.

In order to prove the pharmacodynamic effect of SMS on liver injury induced by HS, we applied SMS to HS-induced BRL-3A cells. It was observed that, compared with the control group, SMS at concentrations ranging from 400 μg/mL to 6400 μg/mL exhibited a dose-dependent proliferative effect on BRL-3A cells, with increasing concentrations showing no significant inhibitory effects (Figure 5A).

In addition, SMS at concentrations between 400 μg/mL and 6400 μg/mL demonstrated a dose-dependent effect in reducing the decrease in BRL-3A cell viability caused by HS (*p* < 0.05) (Figure 5B). Based on these findings, we selected doses of 400, 800, and 1600 μg/mL of SMS, which had significant modulating effects on BRL-3A cell viability under HS, as the low, medium, and high doses for subsequent experiments. A positive control drug, 5 mM NAC, was also included for validation experiments. Meanwhile, we examined the changes in BRL-3A cell viability under HS with interventions of high, medium, and low doses of SMS, as well as 5 mM NAC. The results showed that the cell viability in the HS model group averaged 56.01%. However, following the interventions, cell viability significantly increased to 76.92%, 67.26%, and 67.52% with high, medium, and low doses of SMS, respectively. In the positive control group with NAC, cell viability averaged 65.53% (*p* < 0.05, Supplementary Figure S1).

From the cell morphology, in the HS group, the number of suspended dead cells increased, with cells becoming wrinkled and deformed. The structural outlines were unclear, the nuclear structure was less prominent, and large blank areas were present due to the diffuse distribution of dead cells. After the intervention with SMS and NAC, the number of suspended dead cells was significantly reduced compared to the HS group. Additionally, the number of viable cells increased significantly compared to the HS group, and the number of heterogeneous dead cells was markedly decreased (Figure 5C). Heat shock protein and biochemical analyses revealed that HS significantly upregulated the mRNA expression of HSP70 and HSP90 in cells (*p* < 0.05) and increased the levels of AST and ALT (*p* < 0.001). These changes were reversed following intervention with SMS and NAC (Figure 5D,E).

### 3.6. Protective Effect of SMS on Oxidative Stress Injury Induced by HS in BRL-3A Cells

**Figure 6:**
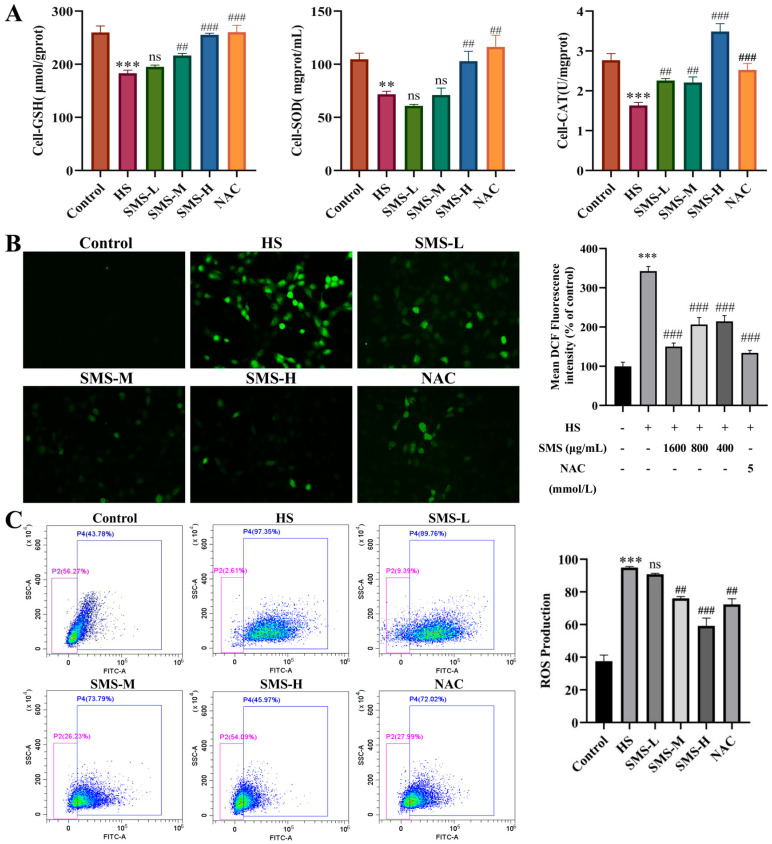
Protective effect of SMS on oxidative stress injury induced by HS in BRL-3A cells. (**A**) Effects of SMS and NAC on GSH, SOD and CAT expression in BRL-3A cells (*n* ≥ 3/group). (**B**) Results of ROS content detected by DCFH-DA probe (*n* ≥ 3/group). (**C**) Results of ROS content detection by flow cytometry (*n* ≥ 3/group). Compared with the control group, ** *p* < 0.01, *** *p* < 0.001; compared with the HS group, ns, not significant, ## *p* < 0.01, ### *p* < 0.001.

To evaluate the intervention effect of SMS on HS-induced oxidative stress in BRL-3A cells, the activities of antioxidant enzymes and the levels of ROS were assessed in this study. Compared with the control group, the levels of GSH, SOD, and CAT in BRL-3A cells were significantly decreased in the HS group (*p* < 0.01). These adverse changes were alleviated following intervention with SMS and NAC, with the high-dose SMS group showing a more pronounced effect (Figure 6A). A fluorescent probe method was used to evaluate the effect of SMS on HS-induced ROS release in BRL-3A cells. Compared with the control group, ROS levels were significantly increased following HS (*p* < 0.01); however, after intervention with SMS or the positive control drug NAC, ROS levels were markedly reduced, with the high-dose SMS group exhibiting the most potent inhibitory effect (Figure 6B). These findings were further confirmed by flow cytometry (Figure 6C).

### 3.7. Effect of SMS on Mitochondrial Dysfunction in HS-Induced BRL-3A Cells

**Figure 7:**
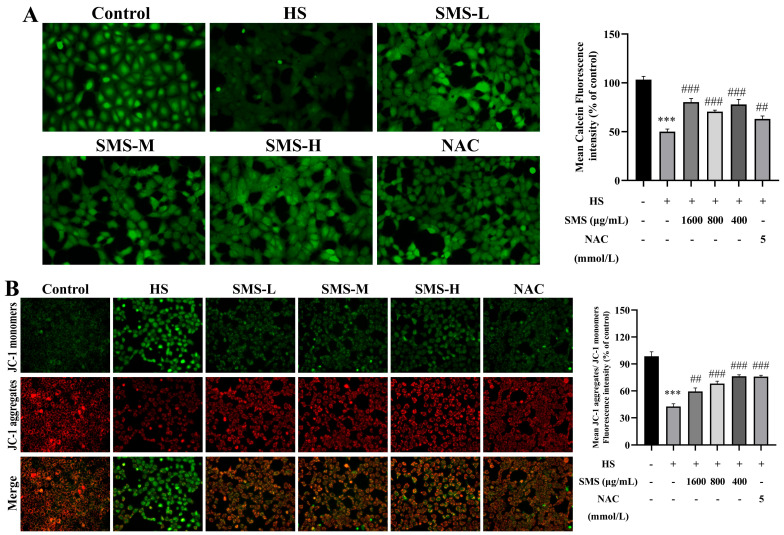
Effect of SMS on mitochondrial dysfunction in HS-induced BRL-3A cells. (**A**) MPTP opening detected by Calcein-AM probe (*n* ≥ 3/group). (**B**) MMP changes, JC-1 aggregates to produce red fluorescence under high mitochondrial membrane potential, whereas it remains as monomers with green fluorescence under low potential (*n* ≥ 3/group). Compared with the control group, *** *p* < 0.001; compared with the HS group, ## *p* < 0.01, ### *p* < 0.001.

To investigate the protective effect of SMS against HS-induced mitochondrial dysfunction in BRL-3A cells, the MMP and the opening of the MPTP were evaluated using a fluorescent probe method. Compared with the control group, HS significantly decreased MMP and induced MPTP opening in BRL-3A cells (*p* < 0.001). Notably, these mitochondrial abnormalities were markedly ameliorated following intervention with SMS and NAC (Figure 7A,B).

## 4. Discussion

As global warming intensifies, the detrimental impacts of HS are expected to become increasingly significant. Clinical and experimental studies have shown that hepatic dysfunction may become more evident during the recovery period after heat exposure, when oxidative imbalance, inflammatory responses, and metabolic disturbances may persist and continue to contribute to tissue injury despite removal of the heat stimulus [10,13,28,29]. In the present study, liver injury was most severe at 6 h in rats and at 9 h in BRL-3A cells, indicating that the post-stress period is a critical stage in hepatic injury progression. In this study, assessments of rat liver histomorphology, serum biochemical markers (ALT and AST), and oxidative stress indicators (GSH, SOD, CAT, T-AOC, and MDA) revealed severe liver injury at 6 h after HS recovery. In vitro experiments further demonstrated that BRL-3A hepatocytes exhibited the most significant damage at 9 h post-HS recovery.

A key finding of this study is that SMS improved multiple aspects of HS-induced liver injury, including histopathological alterations, aminotransferase release, heat shock responses, and oxidative imbalance. This pattern supports a multi-target protective effect and is consistent with the traditional and modern pharmacological application of SMS in heat-related and stress-associated disorders [21,23], as well as with previous studies showing that SMS or SMS-derived preparations alleviate oxidative injury and protect tissues through regulation of energy metabolism, mitochondrial dynamics, and stress-related pathways [14,30,31]. Compared with previous reports, the present study extends the evidence for the beneficial effects of SMS in HS-induced liver injury and suggests that these effects may be associated with restoration of antioxidant capacity and preservation of mitochondrial function. However, SMS did not completely reverse HS-induced injury, suggesting that its action is protective and modulatory rather than absolute.

SMS can inhibit HS-induced oxidative stress injury in the liver by up-regulating the levels of SOD, CAT, GSH, and T-AOC while down-regulating MDA levels. These findings are consistent with previous studies indicating that SMS and its constituent components (such as ginsenosides, ophiopogonins, and schisandra lignans) exhibit significant antioxidant activity in various pathological models [32,33,34,35]. However, oxidative stress in HS is closely linked to mitochondrial dysfunction, disturbed energy metabolism, and cell death signaling [17,18,36,37]. Therefore, the improvement in mitochondrial morphology, stabilization of mitochondrial membrane potential, and inhibition of MPTP opening observed in this study suggest that SMS may protect hepatocytes not only by reducing oxidative burden but also by limiting the interaction between ROS accumulation and mitochondrial injury. Notably, under heat stress conditions, mitochondria serve as the primary source of ROS production, and the scavenging effect of SMS on ROS observed in this study suggests that it may preserve mitochondrial function to maintain cellular energy homeostasis. In addition, the reduced expression of HSP70 and HSP90 after SMS treatment may reflect attenuation of cellular stress, but these findings are not sufficient to identify them as direct molecular targets of SMS.

Mitochondria are particularly vulnerable to oxidative stress, and their functional abnormalities are closely linked to cellular oxidative injury [18,37]. In rats, HS induced mitochondrial swelling and cristae disruption, whereas SMS improved mitochondrial ultrastructure. In BRL-3A cells, SMS also stabilized mitochondrial membrane potential and inhibited MPTP opening after HS exposure. Together, these findings suggest that the protective effects of SMS are associated, at least in part, with preservation of mitochondrial integrity. Since mitochondrial dysfunction contributes to ATP depletion, ROS overproduction, and activation of cell death pathways [38,39,40,41,42], maintenance of mitochondrial homeostasis may be one mechanism involved in the hepatoprotective effects of SMS.

This study has several limitations. First, although the findings support the involvement of oxidative stress and mitochondrial dysfunction in HS-induced liver injury, the causal relationship between these processes was not directly verified. Second, the present study mainly relied on phenotypic, biochemical, and organelle-level observations, and the downstream molecular pathways related to antioxidant regulation, mitochondrial quality control, and cell death remain unclear. Third, HSP70 and HSP90 were evaluated as stress-related indicators, but their mechanistic contribution to the effects of SMS was not examined. Finally, the in vivo experiments were performed only in rats, which may limit direct extrapolation of the present findings to other animal species. Further studies are required to identify the major active constituents of SMS, clarify its key molecular targets, and validate its protective effects in additional animal models under HS conditions.

## 5. Conclusions

In summary, this study demonstrates that SMS protects against HS-induced liver injury through coordinated regulation of oxidative stress and mitochondrial homeostasis. In vivo, SMS ameliorated histopathological damage, reduced serum aminotransferase levels, downregulated the expression of heat shock proteins HSP70 and HSP90, and enhanced hepatic antioxidant capacity. In vitro mechanistic investigations further revealed that SMS improved cell viability, reduced mitochondrial ROS production, stabilized MMP, and inhibited MPTP opening in heat-stressed BRL-3A hepatocytes. Collectively, these findings provide novel insights into the molecular basis underlying the traditional Chinese medicine concept of “replenishing qi and nourishing yin” attributed to SMS, and position this formula as a promising therapeutic candidate for the management of HS-related liver injury.

## Data Availability

The datasets used and/or analyzed during the current study are available from the corresponding author upon reasonable request.

## Supplementary Materials

The following supporting information can be downloaded at: https://www.mdpi.com/article/10.3390/ani16091391/s1, Figure S1: Effects of different concentrations of Sheng Mai San and NAC on the viability of BRL-3A cells induced by heat stress. Table S1: Primer sequences for qRT-PCR.

## Abbreviations

The following abbreviations are used in this manuscript:

HS	Heat stress
SMS	Sheng Mai San
HSP	Heat shock protein
ROS	Reactive oxygen species
MPTP	Mitochondrial permeability transition pore
MMP	Mitochondrial membrane potential
ALT	alanine aminotransferase
AST	aspartate aminotransferase
SOD	superoxide dismutase
MDA	malondialdehyde
GSH	glutathione
CAT	catalase
T-AOC	total antioxidant capacity
NAC	N-acetylcysteine
qPCR	Real-time Quantitative polymerase chain reaction
