# Supplementary material for: Sheng Mai San Regulating the Oxidative Stress and Mitochondrial Damage to Alleviate Liver Injury in Heat Stress Rats
Source: Animals (Basel). 2026 May 2;16(9):1391. doi: 10.3390/ani16091391 (PMC13163120; doi:10.3390/ani16091391)
Supplement: Supplementary file 1 [file animals-16-01391-s001.zip › animals-4256980-supplementary.pdf]

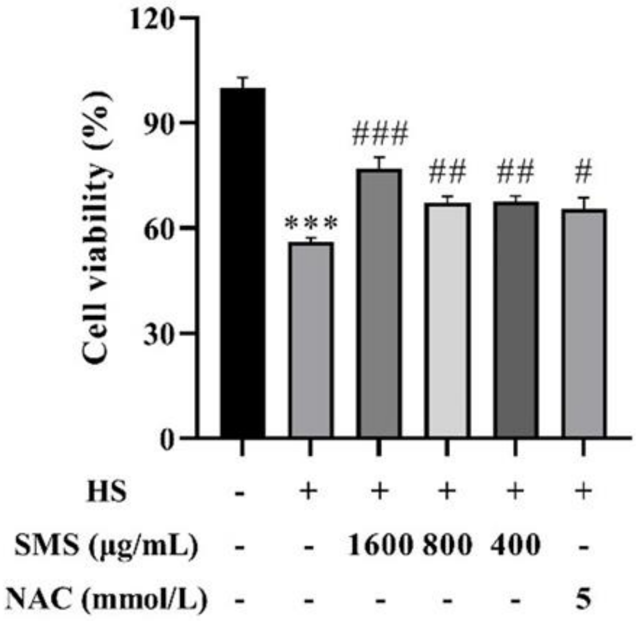

Figure S1. Effects of different concentrations of Sheng Mai San and NAC on the viability of BRL-3A cells induced by heat stress

Table S1. Primers sequences for qRT-PCR

Gene name	Sequence (5′ → 3′)
HSP70	F: TCGAACAGTTCAAGATCAAACG
	R: CCGGAACTTCTTGTAGTAGTCA
HSP90	F: GAAGTGAACAAGCTTGATGGAG
	R: GTCCACGCTATTTTCATCAGAC
GAPDH	F: ACGGGAAACCCATCACCATC
	R: CTCGTGGTTCACACCCATCA
